# Instantaneous dose rate as a crucial factor in reducing mortality and normal tissue toxicities in murine total-body irradiation: a comparative study of dose rate combinations

**DOI:** 10.1186/s10020-025-01135-3

**Published:** 2025-02-26

**Authors:** Hongyu Zhu, Shihua Liu, Jiaqi Qiu, Ankang Hu, Wanyi Zhou, Jian Wang, Weihang Gu, Yinuo Zhu, Hao Zha, Rong Xiang, Junli Li, Rui Qiu, Chong Zhao, Peng Huang, Xiaowu Deng

**Affiliations:** 1 Department of Radiation Oncology, State Key Laboratory of Oncology in South China, Collaborative Innovation Center for Cancer Medicine, Sun Yat-sen University Cancer Center, Yuexiu District, Guangzhou City, 510060 Guangdong province China; 2 United Laboratory of Frontier Radiotherapy Technology of Sun Yat-sen University & Chinese Academy of Sciences Ion Medical Technology Co., Ltd, Guangzhou, 510060 China; 3 State Key Laboratory of Oncology in South China, Collaborative Innovation Center for Cancer Medicine, Sun Yat-sen University Cancer Center, Guangzhou, 510060 China; 4 Beijing Huaqingjia High Energy Electron Technology Corporation Limited, Beijing, 100091 China; 5 Department of Engineering Physics, Tsinghua University, Beijing, 100084 China; 6 Department of Nasopharyngeal Carcinoma, State Key Laboratory of Oncology in South China, Collaborative Innovation Center for Cancer Medicine, Sun Yat-sen University Cancer Center, Guangzhou, 510060 China

## Abstract

**Purpose:**

The ultra-high dose rate (UHDR) radiation shows promise in eradicating tumors while reducing normal tissue toxicities. However, the biological outcomes of UHDR are influenced by various factors, particularly the mean dose rate and instantaneous dose rate. Additionally, the UHDR response at large field sizes is lacking. This study aimed to explore the impact of different dose rate combinations on gastrointestinal biological outcomes following total-body irradiations (TBI) and to examine the involved molecular signaling pathways.

**Method:**

Female C57BL6/J mice received 10 Gy TBI using three modes: ultra-high mean and ultra-high instantaneous dose rate irradiation (HH mode), low mean and ultra-high instantaneous dose rate irradiation (LH mode), and low mean and low instantaneous dose rate irradiation (LL mode). Mice were euthanized at 3 h and 48 h post irradiation to assess acute normal tissue damage and perform transcriptome sequencing. Furthermore, a subset of mice was monitored for 30 days to evaluate survival.

**Results:**

We found that when the instantaneous dose rate is sufficiently high (> 10^5^ Gy/s), both ultra-high or low mean dose rate irradiation reduced mice mortality, myelosuppression, DNA damage, and cell apoptosis. The survival probabilities 30 days after 10 Gy TBI were 4/7, 4/6, and 0/6 in the HH, LH, and LL groups, respectively. Myelosuppression was lower at 3 h and 48 h post HH and LH irradiations than LL irradiation. The better regulated inflammatory response was evident at 48 h post HH and LH irradiation compared to LL irradiation. Additionally, DNA damages and cell apoptosis in the intestinal tissue were significantly reduced after HH and LH irradiations compared to LL irradiation. Transcriptome sequencing of intestinal tissues revealed that HH irradiation activated immune response pathways and suppressed mitochondrial related pathways compared to LL irradiation.

**Conclusion:**

Our findings underscore the pivotal role of instantaneous dose rate in reducing radiation damages. When the instantaneous dose rate is sufficiently high (> 10^5^ Gy/s), both ultra-high or low mean dose rate irradiation (HH and LH mode) reduced mice mortality, myelosuppression, DNA damage, and cell apoptosis. Understanding these dose rate effects and biological responses are crucial for optimizing radiotherapy strategies and exploring the potential benefits of UHDR irradiation.

**Supplementary Information:**

The online version contains supplementary material available at 10.1186/s10020-025-01135-3.

## Introduction

It has been reported that ultra-high dose rate (UHDR) irradiation could effectively eradicate tumors while mitigating radio-toxicities in normal tissue compared to conventional dose rate (CONV) irradiation (Favaudon et al. 2014). The unique biological response of UHDR is known as FLASH effect and hascaused widespread attention, and its tumor-killing effect and normal tissue sparing effects was successfully demonstrated in a variety of models with different endpoints (Favaudon et al. 2014; Gao et al. 2022; Shi et al. 2022; Zhu et al. 2022; Levy et al. 2020; Montay-Gruel et al. 2017, 2019). Now the preclinical research and clinical translation of UHDR radiation is being actively promoted.

However, the biological outcome of UHDR radiation is affected by many factors, including mean dose rate, instantaneous dose rate, dose fractionation, pulse number, pulse separation, delivery time, etc. (Vozenin et al. 2020). Typically, UHDR radiation experiments have been conducted under conditions of both ultra-high mean and ultra-high instantaneous dose rates (Favaudon et al. 2014; Vozenin et al. 2019; Chaudhary et al. 2023; Tinganelli et al. 2022; Tessonnier et al. 2021). Ruan et al. reported that the intestinal crypt sparing effect was observed with mean dose rate > 280 Gy/s and the effect was lost with increased dose delivery time (Ruan et al. 2021). Review data from Montay-Gruel et al. show that the dose rate in pulses should be > 1 × 10^5^ Gy/s and the delivery time for 10 Gy should be within 1s to obtain the sparing effect (Montay-Gruel et al. 2021). Whether the mean dose rate or the instantaneous dose rate is the decisive factor for the FLASH effect remains an unanswered question. Determining this is crucial for the clinical translation and medical equipment development of UHDR radiation.

Nearly all of the preclinical studies on the effects of UHDR radiation focused on localized irradiation with relatively small field sizes, for brain (Montay-Gruel et al. 2017, 2019; Allen et al. 2023; Dokic et al. 2022), thorax (Favaudon et al. 2014; Kim et al. 2021), abdomen (Shi et al. 2022; Levy et al. 2020; Ruan et al. 2021; Diffenderfer et al. 2020), and skin (Soto et al. 2020; Cunningham et al. 2021). With the reduced normal tissue toxicity of UHDR radiation, larger irradiation fields or total-body irradiation (TBI) might be considered to improve tumor control, or mitigate normal tissue toxicities in the context of preconditioning for hematopoietic stem cell transplantation (HSCT) (Hoeben et al. 2021) or palliative treatment of disseminated malignancies (Quast 2006). While large field FLASH has been explored to some extent, the biological response under these conditions remains poorly studied. For example, the FAST-01 and FAST-02 clinical trials utilized field sizes from 7.5 cm × 7.5 cm to 7.5 cm × 30 cm (Daugherty et al. 2023, 2024), Konradsson et al. treated canine cancer patients with various field sizes, from a 2 cm in diameter up to the largest rectangular field size of 8 cm × 4cm^25^. These limited field sizes hinder our ability to draw definitive conclusions of application potential of UHDR radiation.

The molecular response to CONV radiation exposure has been well characterized, while the mechanism of FLASH effect is still elusive and need to be elucidated. The key hypotheses of FLASH sparing effect include oxygen depletion, reduced reactive oxygen species (ROS), alterations in the immune microenvironment, preservation of DNA integrity, pH regulation, and modulation of mitochondrial function, etc. (Shi et al. 2022; Liu et al. 2023a, b; Limoli and Vozenin 2023). A better understanding of the FLASH effect and the possibility of controlling its occurrence will facilitate meaningful clinical implementation (Vozenin et al. 2022).

In this study, we performed TBI with a murine model and employed a combination of different dose rates, that is, the ultra-high mean dose rate and ultra-high instantaneous dose rate irradiation (HH mode), low mean dose rate and ultra-high instantaneous dose rate irradiation (LH mode), and low mean dose rate and low instantaneous dose rate irradiation (LL mode). Our objective was to determine whether the mean dose rate or instantaneous dose rate is the critical factor for the FLASH effect, and to assess the mortality, hematopoietic and gastrointestinal (GI) normal tissue toxicity across different irradiation modes. Transcriptome sequencing was utilized to analyze the mRNA levels in the intestine to screen the differential signaling pathways activated upon LL and HH modes. We set out to clarify the important role of mean dose rate and instantaneous dose rate in the normal tissue sparing effect, and clarify the differentially hematopoietic and GI biological response and molecular signaling pathway between LL and HH modes, which provides mechanistic insights into the physiological changes.

## Methods and materials

### Animal model

Female C57BL6/J mice, aged 7–8 weeks, were purchased from Gempharmatech Co., Ltd (Jiangsu, China) and allowed to acclimate for 1 week before irradiation. All mouse experiments adhered to the approved guidelines of the Institutional Animal Care and Use Committee of Sun Yat-sen University Cancer Center (L102042022060C) and Tsinghua University (22-QR1). Mice were anesthetized with avertin (intraperitoneal injection,15 µl/g), and received 10 Gy electron TBI and were euthanized at 3 h and 48 h post irradiation to determine acute normal tissue damage, or followed up for 30 days to observe survival status.

### Irradiation setup

Three different dose rate combinations were employed in this study, namely: (1) HH mode is the ultra-high mean dose rate and ultra-high instantaneous dose rate mode, typically used in UHDR experiments. (2) LH mode is low mean dose rate and ultra-high instantaneous dose rate mode. (3) LL mode is the low mean dose rate and low instantaneous dose rate mode, commonly utilized in clinical radiotherapies. Table 1 provides the specific beam characteristic details for each of these three irradiation modes. The HH and LH modes of irradiations were performed using a compact linear accelerator and the LL mode of irradiations were performed using a clinical Elekta Infinity linac (Elekta AB, Stockholm, Sweden).

The compact linear accelerator generates a beam current of approximately 53 mA, which is not adjustable. Therefore, the adjustment of dose rate is achieved by adjusting the source surface distance (SSD), which can be modified by repositioning the electron collimator and the sample platform. This adjustment allows for the alteration of the dose rate to either UHDR or CONV levels.

**Fig. 1:**
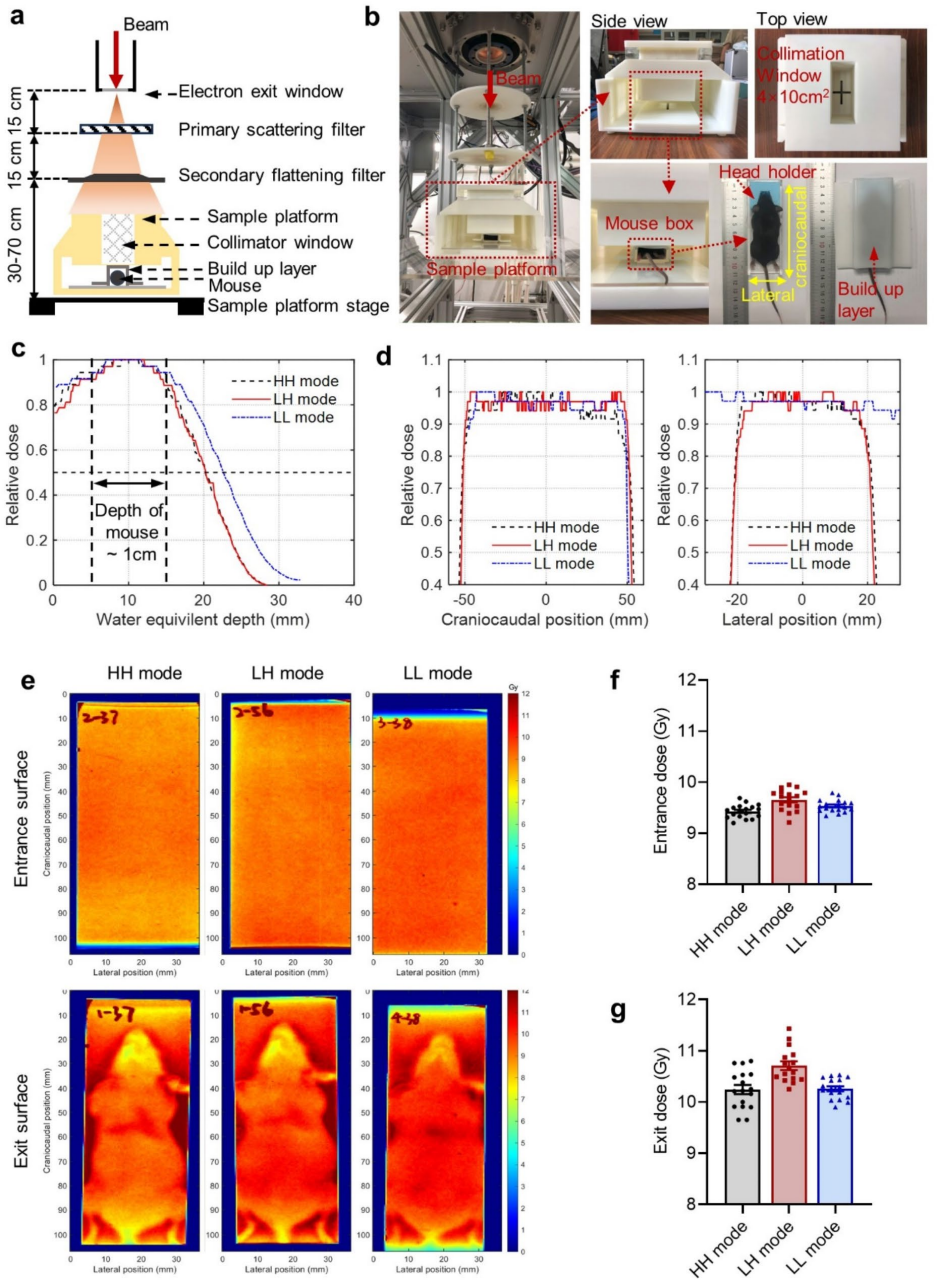
Experimental setup and parameters for HH, LH, and LL modes. (**a**) A schematic drawing of the HH and LH irradiation set-up. (**b**) Photo of the 3D-printed assembly and mouse box adopted in HH and LH irradiation. (**c**) Central axis percentage depth dose (PDD) of HH, LH, and LL modes of irradiation. (**d**) Relative dose profile measured with EBT3 films at the craniocaudal (left) and lateral (right) central axis at the entrance surface. (**e**) Representative dose distribution measured with EBT3 films. (**f**) Entrance and (**g**) exit doses of each irradiated mice measured with EBT3 films

The HH and LH mode beams were flattened to acquire acceptable beam flatness and penumbra at large field size. Briefly, a beam shaping system has been integrated into the platform (Fig. 1a and b). This system comprises a primary scattering filter, a secondary flattening filter, and a collimator, all constructed from acrylonitrile butadiene styrene (ABS) material and aligned in a vertical configuration. The primary scattering filter is a thin disc that initially expands the electron beam emitted from the titanium window, thereby achieving a larger potential radiation field size. The secondary flattening filter incorporates elements of a conical frustum and a disc, and is intended to reduce the intensity of the electron beam in the central area while enhancing the uniformity of the radiation field. The collimator, with sufficient thickness, prevents electron penetration and further improves flatness within the field by scattering electrons through its apertures. The size parameters of these structures determine the final beam energy, the field size, flatness and penumbra, which are optimized using the Monte Carlo method.

The field sizes adopted in the HH, LH, and LL modes were 4 × 10 cm^2^, 4 × 10 cm^2^ and 10 × 10 cm^2^ these fields were large enough to cover the whole body of mice (excluding the mice tail). A 3D-printed assembly (Fig. 1a and b) was used as a sample platform and electron collimator during the HH and LH mode irradiation, and a custom-made mice box (Fig. 1b) was employed for mouse positioning. Water equivalent buildup layer with a thickness of 4.8 mm was used during irradiation. Dose delivery on the compact linear accelerator was controlled by adjusting pulse number with a pulse number counter in a programmable logic controller, and this method was validated on the previously reported X-ray UHDR platform, which demonstrated a dose delivery instability of < 5% Liu et al. 2023. In addition, the absolute dose received by each mouse was measured with GAFChromic EBT3 films (Ashland Inc., Covington, KY, USA), which were attached to the back and chest abdominal surface of each mouse, and the entrance and exit electron doses were determined from the center region of the films. The dose delivery was prescribed to the entrance surface of the mice to achieve 10 Gy. However, in HH mode, the dose per pulse limited the actual dose received at the entrance surface to 9.4–9.5 Gy with 5 HH pulses. To minimize the relative dose difference among the three modes, the entrance doses of the LH and LL modes were adjusted accordingly. Additionally, the percentage depth dose (PDD) distribution was measured with EBT3 film, which was placed in 3D-printed assembly for HH and LH modes, and in solid water for LL mode. The beam field sizes used in the PDD measurements were the same as those use for mice irradiation. The films were positioned at the field center and aligned parallel to the beam direction. The mean energy of electron beam can be calculated following the relationship of $\:\stackrel{-}{E}$=2.33×R_50_, where $\:\stackrel{-}{E}$ is the mean energy (MeV) and R_50_ is the water equivalent depth in cm of the 50% dose level (Andreo et al. 1987).

**Table 1:** Irradiation parameters

	Unit	HH mode	LH mode	LL mode
Irradiator		A compact linear accelerator		Elekta Infinity linac
Field size	cm^2^	4 × 10	4 × 10	10 × 10
Source surface distance (SSD)	cm	60	100	95
Pulse repetition frequency	Hz	250	1	300
Pulse width (Full with at half maximum)	µs	3.92	3.92	3
Mean dose rate	Gy/s	468	0.4	0.1
Instantaneous dose rate	Gy/s	4.78 × 10^5^	1.02 × 10^5^	111
Mean electron energy	MeV	4.7	4.7	5.3
Total delivery time	s	0.02	24	100
Number of pulses		5	24	28,600
Dose per pulse*	Gy	1.89–1.90	0.399–0.401	0.00033
Entrance Dose (mice)**	Gy	9.428 ± 0.131	9.655 ± 0.206	9.534 ± 0.122
Exit dose (mice)**	Gy	10.24 ± 0.371	10.71 ± 0.334	10.26 ± 0.200

* Measured at the entrance surface

** Mean ± standard deviation (SD)

It should be noted that the scatter angle of electron beams was changed after passing the primary scattering and secondary flattening filter and the “virtual source position” moved to a downstream direction, which results in an altered “virtual SSD”. Therefore, the instantaneous dose rates of the HH and LH modes do not follow the inverse square principle.

### Whole blood count and cytokine detection

Peripheral blood from the eyeball was collected into an EDTA-coated capillary at the time of euthanasia. Complete blood count data were analyzed using the BC-2800Vet auto hematology analyzer (Mindray, Shenzhen, China). Serum was collected from the supernatant of peripheral blood after 10 min centrifugation at 3000×g and stored at − 80 °C until cytokine measurement. Pro-inflammatory cytokines, including tumor necrosis factor alpha (TNF-α) and interleukin 6 (IL-6), as well as the anti-inflammatory cytokine IL-10, were quantified using enzyme-linked immunosorbent assay (ELISA) kits (Elabscience, Wuhan, China, Cat. Nos.: E-MSEL-M0001, E-MSEL-M0002, and E-MSEL-M0031), following the manufacturer’s instructions.

### Immunohistochemistry staining, Terminal deoxynucleotidyl transferase dUTP nick end labeling (TUNEL) assay and image analysis

The intestinal tissues were fixed in 10% neutral buffered formalin and processed for paraffin embedding using the Swissroll technique. Slices of the embedded intestinal tissues were cut at a thickness of 4 μm. Immunohistochemical (IHC) staining was conducted on the intestinal tissue sections to assess DNA damage with Phospho-Histone H2A.X (Ser139) (CST, Massachusetts, Cat. No.: 80312 S, 1:100 dilution) and changes in the number of proliferating cells were determined using the Ki-67 antibody (Abcam, Cambridge, Cat. No.: ab15580, 1:100 dilution). The terminal deoxynucleotidyl transferase dUTP nick end labeling (TUNEL) assay kit (Promega, Madison, Cat. No.: G3250) was employed to identify apoptotic cells in intestinal tissues, following the manufacturer’s instructions.

The IHC and TUNEL sections were scanned using a KF-PRO-020 whole slide scanner (KFBIO, Ningbo, China) and subsequently analyzed using the HALO image analysis platform (v3.6.4134.193, Indica Labs, Albuquerque, NM, USA). The Multiplex IHC v3.1.4 and CytoNuclear FL v2.0.12 algorithms were adopted and modified for IHC and TUNEL section analyses, respectively. The nuclear counterstain was used as reference to detect and segment cells, and signal thresholds were adjusted for each channel to assess cell positivity, i.e., negative, moderate positive, and strong positive. The number of moderate positive (N_mp_) and strong positive cells (N_sp_) were defined, and the final positive index was calculated as follows: positive index = N_mp_ + 2×N_sp_ for evaluation. Further detailed descriptions are provided in the supplementary material (see example Fig. S1).

### RNA extraction, library construction and sequencing

Transcriptome sequencing was used to find alterations in signal transduction pathways evoked by different irradiation modes. Total RNA of intestine tissue was extracted using TRIzol reagent kit (Invitrogen, Carlsbad, CA, USA) according to the manufacturer’s protocol. mRNA was subsequently enriched by Oligo (dT) beads, followed by fragmentation into short fragments using fragmentation buffer. Reverse transcription into cDNA was performed using NEBNext Ultra RNA Library Prep Kit for Illumina (NEB#7530, New England Biolabs, Ipswich, MA, USA). The purified double-stranded cDNA fragments were end repaired, A base was added, and ligated to Illumina sequencing adapters. The ligation reaction was purified with the AMPure XP Beads (1.0×) and subjected to polymerase chain reaction (PCR) amplification. The resulting cDNA library was sequenced using Illumina Novaseq6000 by Gene Denovo Biotechnology Co. (Guangzhou, China).

### Quantification and differential expression analysis

Quantification of the transcripts and genes was performed using StringTie v1.3.1 software, and fragments per kilobase of transcript per million mapped reads (FPKM) values were obtained. RNA differential expression analysis was performed by DESeq2 software between two different groups (and by edgeR between two samples). Genes with absolute value of log_2_ (fold change) > 1 (|log_2_FC|>1) and false discovery rate (FDR) < 0.05 were identified as differentially expressed.

### Gene enrichment and immune cells infiltration analysis

Metascape (https://metascape.org/) was used for enriching the functions of differentially expressed genes. CIBERSORTx (https://cibersortx.stanford.edu/) was utilized for analyzing the immune cell infiltration. First, the IDs of mouse genes were converted into human genes and the duplicate genes were deleted. Next, the “Impute cell fractions” analysis utilized the signature matrix file “LM22 (22 immune cell types)”and the gene counts for mixture file, with quantile normalization disabled (recommended for RNA-seq).

### Deposit of transcriptome data in public database

The transcriptome sequencing data were deposited in the Gene Expression Omnibus (GEO) database: https://www.ncbi.nlm.nih.gov/geo/ under accession number GSE271654.

### Statistical analyses

Statistical analyses were conducted using Prism version 8.4.0 (GraphPad software). Group comparisons among HH, LH, and LL groups were performed using one-way analysis of variance (ANOVA), followed by Tukey’s multiple comparisons post-hoc test. Survival probabilities were depicted using Kaplan–Meier curves and assessed with log-rank test analysis. Student’s t-test was employed for comparisons between two groups. All data are expressed as mean ± standard error of the mean (SEM).

## Results

### Dosimetry

HH and LH mode irradiation were performed using a compact linear accelerator with well-established dosimetric characteristics, as described in previous publications (Liu et al. 2023a, b). The 3D-printed assembly and custom-made mice box used during the HH and LH mode irradiation are shown in Fig. 1a, b. The percentage depth dose (PDD, Fig. 1c) and dose profile (Fig. 1d) of HH, LH and LL modes irradiation were measured with EBT3 film. The irradiation beams for HH, LH and LL modes were all flattened (Fig. 1d), and the representative dose distribution measured with EBT3 films were presented in the Fig. 1e. The entrance and exit dose of each irradiated mouse measured with EBT3 films is shown in Fig. 1f- g as well as Table 1.

### Ultra-high instantaneous dose rate irradiation caused better survival and lower myelosuppression

**Fig. 2:**
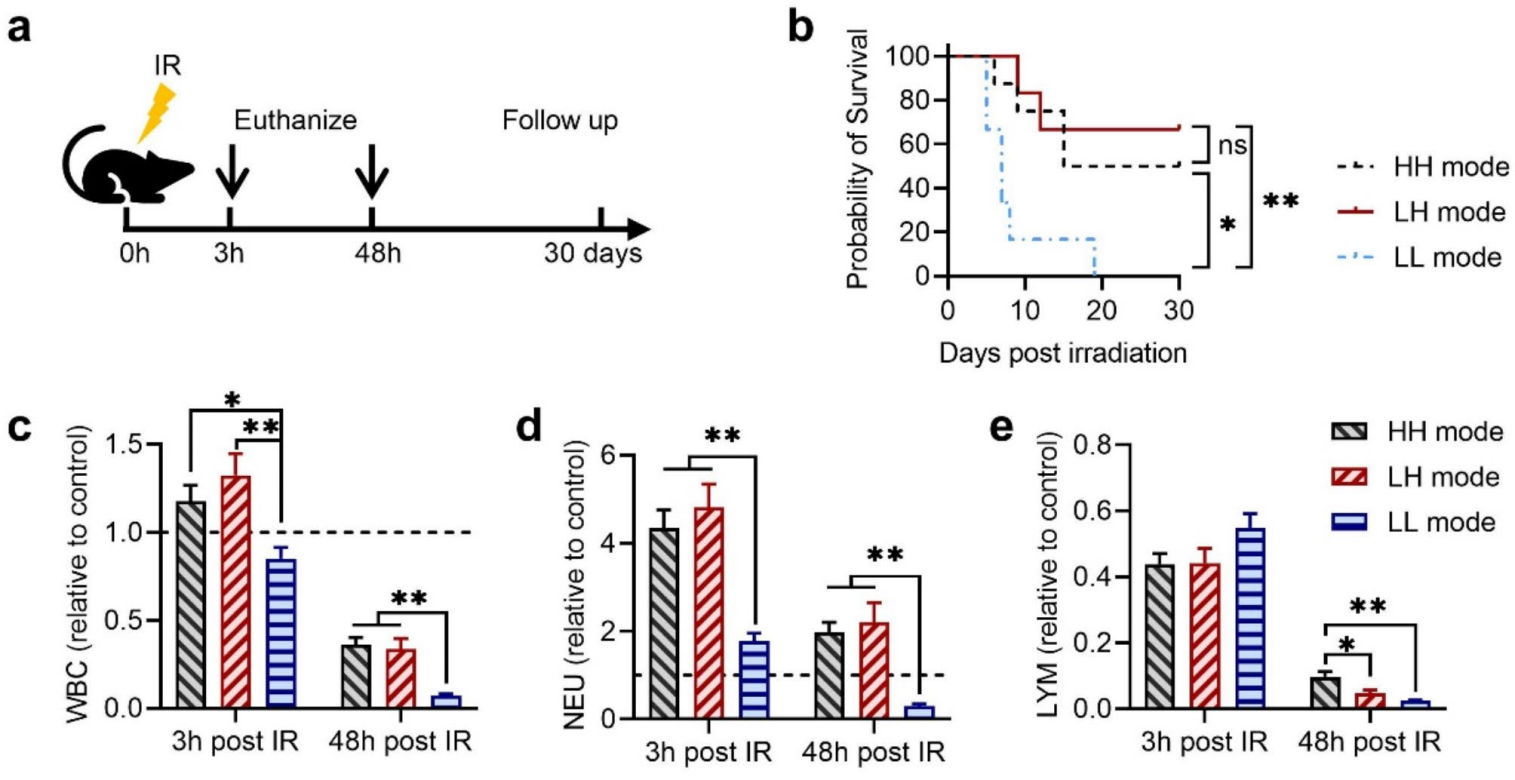
The probability of animal survival and blood counts after total-body irradiation (TBI). (**a**) Schematic overview of the study design. (**b**) Kaplan–Meier curve of post TBI overall survival. (**c**) - (**e**) Blood counts of white blood cells (WBC) (**c**), neutrophils (NEU) (**d**), and lymphocytes (LYM) (**e**) at 3 h and 48 h post TBI. Data are presented as blood cell counts relative to the control group. *n* ≥ 5 in each group, *p < 0.05, **p < 0.01, ***p < 0.001. Bars represent mean ± SEM

To compare toxicities and mortality of 10 Gy TBI at different dose rates, mice received HH, LH, and LL mode irradiation, and were euthanized at 3 h and 48 h post-irradiation to determine acute toxicities, or followed up for 30 days to track the mortality (Fig. 2a). The survival probabilities after 10 Gy TBI were 4/7, 4/6, and 0/6 in the HH, LH, and LL mode groups, respectively (Fig. 2b). Significantly reduced mortalities were observed in the HH and LH mode groups (ultra-high instantaneous dose rate) compared to the conventional LL mode irradiation.

Besides, peripheral blood was collected at 3 h and 48 h post-irradiation for blood counts, and the changes of blood cell count relative to the control are shown in Fig. 2c-e. Previous studies based on murine or ferret modes have shown that the acute effects of TBI on white blood cell and lymphocyte counts follow a dose-dependent decrease manner (Ware et al. 2010; Sanzari et al. 2013), while the neutrophil count increase at 3 h post irradiation and decrease at 48 h post irradiation (Sanzari et al. 2013). Our result shows at 3 h post-irradiation, white blood cell counts in the HH and LH groups were significantly higher than that of conventional LL group (Fig. 2c). Additionally, at 3 h post-irradiation, the neutrophil counts significantly increased in all HH, LH and LL groups (Fig. 2d), while the lymphocyte counts in these groups were approximately half of the control level. (Fig. 2e). It is conceivable that the pronounced increase in neutrophil may have partially offset the decrease of lymphocyte when determining the overall white blood cell count. At 48 h post-irradiation, the white blood cell counts in the HH, LH, and LL groups were only 0.36, 0.34, and 0.07 times that of the control group, respectively (Fig. 2c). Additionally, the counts of neutrophil and lymphocyte in the LL mode group are substantially lower compared to the HH and LH mode groups (Fig. 2d, e). This suggests that the conventional LL mode irradiation induced more severe myelosuppression, which could be resulted from a higher equivalent dose compared to HH and LH mode irradiation.

### Ultra-high instantaneous dose rate irradiation induced fierce pro-inflammatory cytokine response and rapid recovery from irradiation

**Fig. 3:**
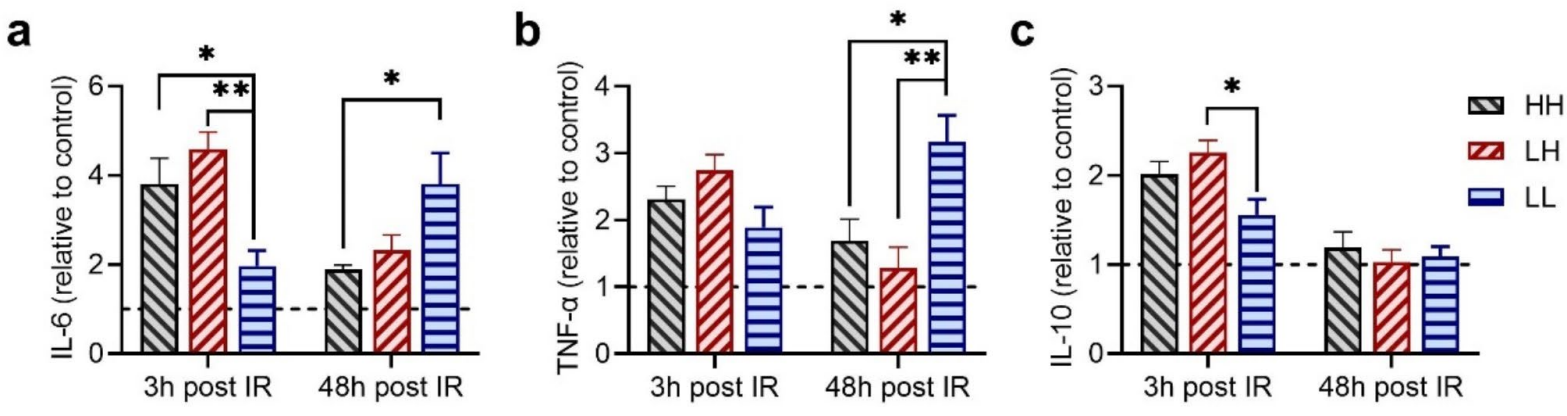
Cytokine responses after different modes of irradiation. (**a**-**c**) Relative change to control of (**a**) interleukin (IL)-6, (**b**) tumor necrosis factor alpha (TNF-α), and (**c**) IL-10 detected by ELISA methods response at 3 h and 48 h post-irradiation. Data are presented as expression level relative to the control group. *n* ≥ 5 in each group, *p < 0.05, **p < 0.01, ***p < 0.001. Bars represent mean ± SEM

TBI can trigger systemic inflammatory reaction, and the serum cytokine profile can serve as a surrogate marker of systemic activation of the immune system (Bell et al. 2019). We collected the serum of peripheral blood at 3 h and 48 h post irradiation for cytokine detection. At 3 h post irradiation, the IL-6 levels in the HH and LH group were ~ 4 times higher relative to control, which were significantly higher than that of the LL mode group (~ 2 times relative to control, Fig. 3a). The TNF-α levels in the HH, LH, and LL groups increased to ~ 2 times relative to control, with no statistic difference observed among the groups (Fig. 3b). The anti-inflammatory cytokine IL-10 increased to ~ 2 times higher relative to control in the HH and LH groups, while a statistically lower level of IL-10 was observed in the LL group (Fig. 3c).

At 48 h post irradiation, the pro-inflammatory cytokines IL-6 and TNF-α in the LL group continued to increase, reaching 3.8 and 3.2 times higher relative to control, respectively (Fig. 3a, b). In contrast, the IL-6 level in the HH and LH group decreased form ~ 4 times higher to ~ 2 times higher relative to control (Fig. 3a). The level of anti-inflammatory cytokine IL-10 levels in all dose rate groups returned to the levels observed in the control group (Fig. 3c).

### Ultra-high instantaneous dose rate irradiation reduced DNA damage and apoptosis in the intestine

**Fig. 4:**
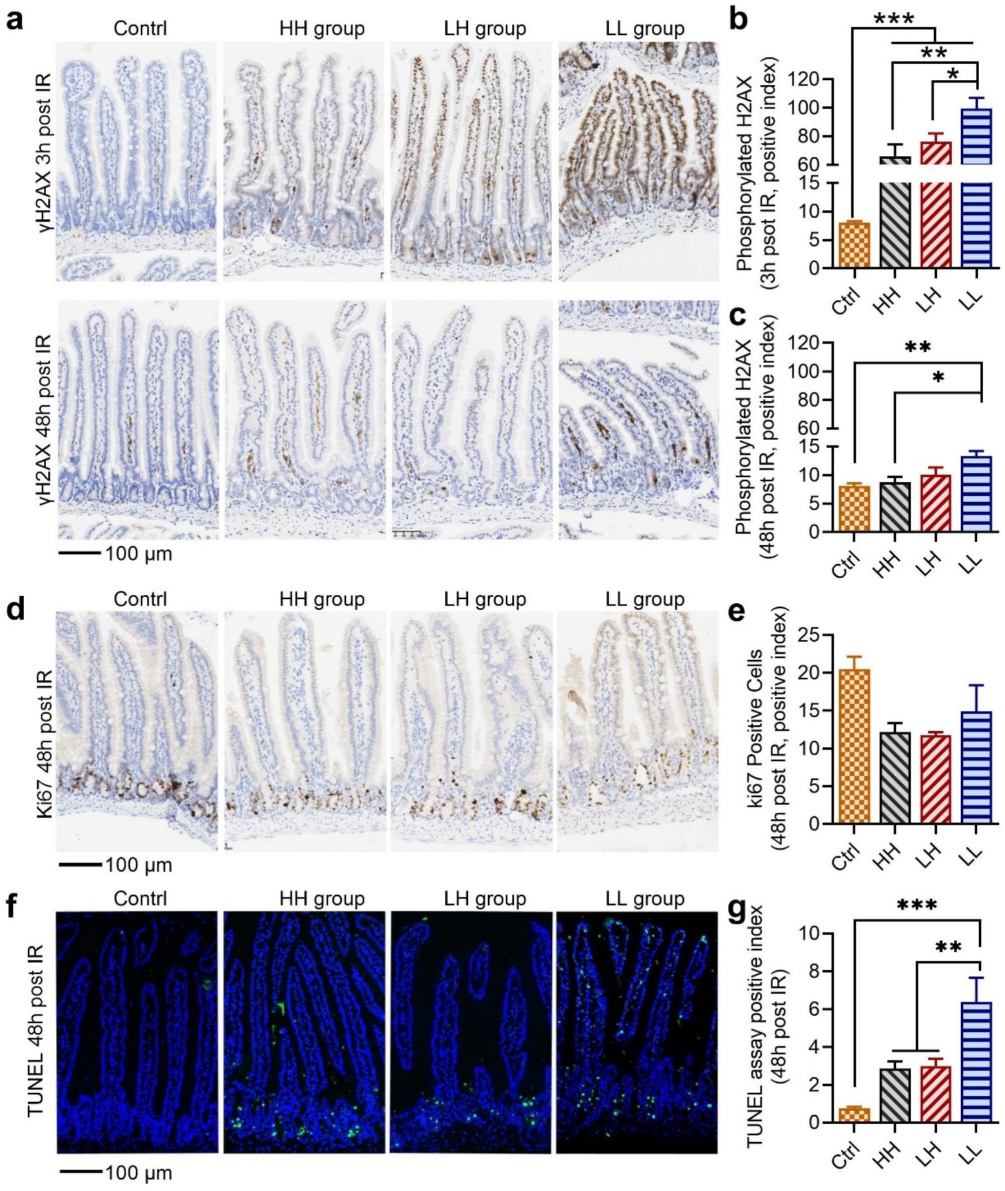
Quantification of tissue damage in the intestine after total-body irradiation (TBI). (**a**) Representative immunohistochemical (IHC) stained γH2AX image of the intestinal tissue at 3 h (upper) and 48 h (bottom) post-irradiation. (**b**) and (**c**) Positive index of γH2AX at 3 h (**b**) and 48 h (**c**) post-irradiation. (**d**) Representative image of IHC stained ki67 of the intestinal tissue at 48 h post-irradiation and (**e**) positive index of ki67 at 48 h post-irradiation. (**f**) Representative image TUNEL assay of the intestinal tissue at 48 h post-irradiation and (**g**) positive index of TUNEL at 48 h post-irradiation. *n* ≥ 5 in each group, *p < 0.05, **p < 0.01, ***p < 0.001. Bars represent mean ± SEM

Tissue damage at 3 h and 48 h post irradiation was quantified with IHC staining of the phospho-Histone H2A.X (Ser139), a surrogate marker for DNA double strand breaks. Our results indicate that all irradiation modalities led to a noticeable increase in DNA damage (Fig. 4a-c). Significantly increased DNA damage was observed at 3 h post conventional LL irradiation compared to HH and LH modes (Fig. 4a upper and **b**). By 48 h post-irradiation, most of the DNA damage had been repaired. The γH2AX index of HH and LH modes had recovered to the control level, while the highest γH2AX index was still observed in the conventional LL group at 48 h post-irradiation (Fig. 4c).

The numbers of proliferating intestinal crypt cells and apoptotic cells were examined at 48 h post-irradiation. A reduction in Ki67^+^ crypt cells was observed after irradiation, although no statistically significant difference was noted among the HH, LH, and LL groups (Fig. 4d, e). Besides, the apoptosis of intestinal cells increased significantly in the LL group compared to the HH and LH groups (Fig. 4f, g). This result was consistent with our previous study (Zhu et al. 2022, Zhu et al. 2019 and result from Levy et al. (Levy et al. 2020) on the abdominal CONV and UHDR radiation.

### HH irradiation activated immune associated responses and inhibited mitochondrial related progresses

**Fig. 5:**
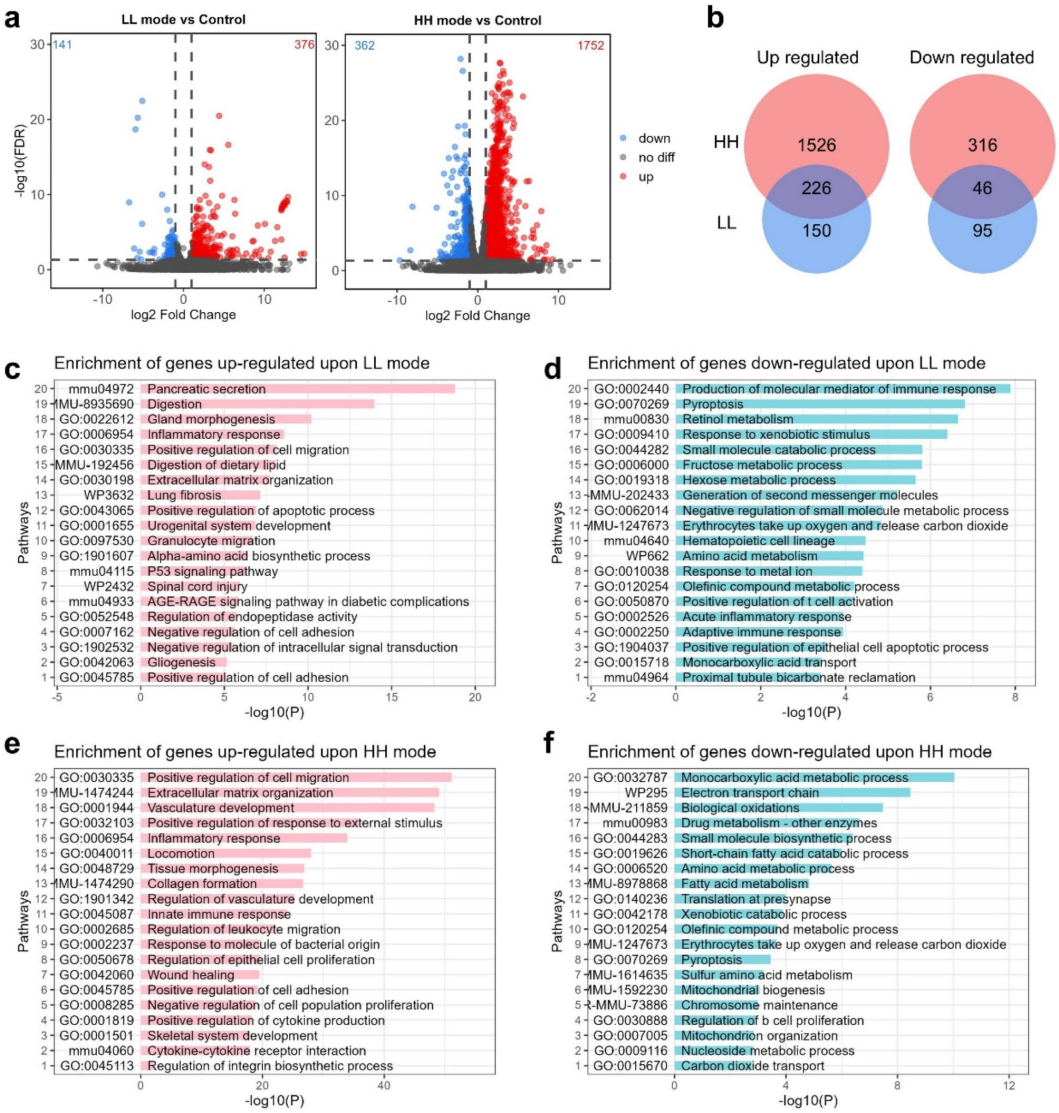
Global changes and enrichment of differential expression genes in the intestine post-LL and HH irradiation. (**a**) Volcano plot of differential expression genes in the TBI model at 48 h post- LL (left) and HH (right). (**b**) Venn diagram of the common and unique genes between the HH and LL irradiation groups. Histogram of the top 20 gene ontology (GO) enrichment terms for significantly upregulated or downregulated genes in LL groups (LL_TBI vs. Control) (**c**, **d**) or HH groups (HH_WBI vs. Control) (**e**, **f**). Date was generated using the Metascape tool

Considering that HH and LH irradiation led to similar biological responses, only the intestinal tissues at 48 h post-LL or HH irradiation were adopted for transcriptome sequencing (supplementary Table 1). We compared the mean expression of mRNA in the LL groups (LL_TBI vs. Control), and found that 376 and 141 mRNAs were upregulated and downregulated (|log_2_FC|>1, and FDR < 0.05), respectively (Fig. 5a and supplementary Table 2). In the HH groups (HH_TBI vs. Control), 1752 and 362 mRNAs were upregulated and downregulated (|log_2_FC|>1, and FDR < 0.05), respectively (Fig. 5a and supplementary Table 3). The Venn diagram illustrates the common and unique genes between the HH and LL irradiation groups (Fig. 5b). Next, we used Metascape for gene ontology (GO) pathway enrichment analysis of the upregulated or downregulated genes after LL irradiation (Fig. 5c-d) or HH irradiation (Fig. 5e-f) compared to their respective control groups. In global analysis, we found that more genes and signaling pathways were activated in HH than in LL groups (Fig. 5a).

**Fig. 6:**
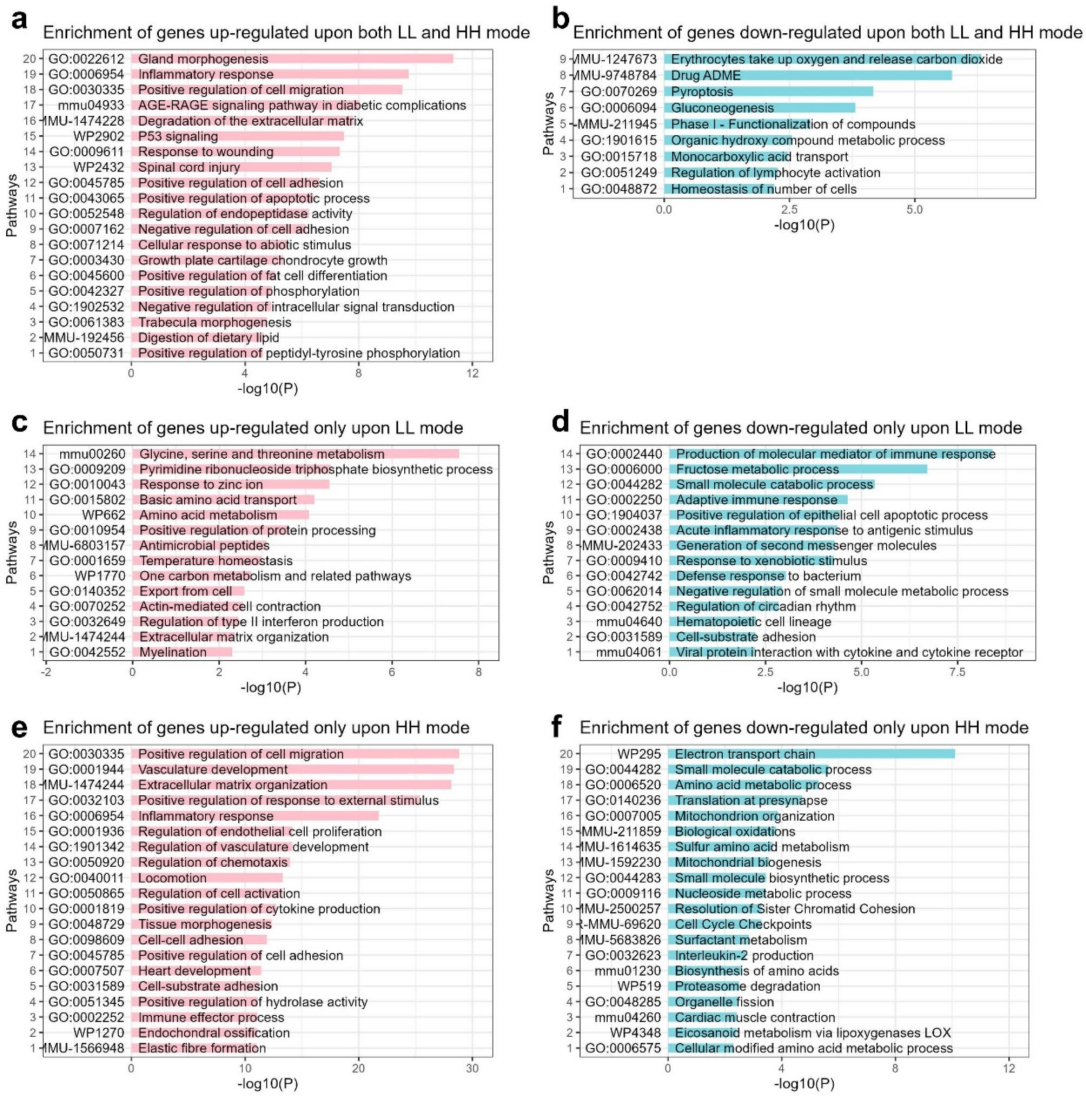
Enrichment of differential expression genes in the intestine post-LL and HH irradiation. Histogram of the top GO enrichment terms for significantly (**a**) upregulated or (**b**) downregulated genes in both LL groups (LL_TBI vs. control) and HH groups (HH_TBI vs. Control). Histogram of the top GO enrichment terms for significantly upregulated or down regulated genes only in LL groups (LL_TBI vs. Control) (**c**, **d**) or only in HH groups (HH_TBI vs. control) (**e**, **f**). Data were generated using the Metascape tool

GO pathway enrichment of upregulated or downregulated genes upon both LL and HH irradiation were analyzed (Fig. 6a and b). The results showed that the signaling pathways of gland morphogenesis, inflammatory response, cell migration et al. were activated (Fig. 6a), while the signaling pathways of erythrocytes take up oxygen and release carbon dioxide, drug ADME, pyroptosis, et al. were inhibited (Fig. 6b). The regulation of lymphocyte activation signal inhibition was consistent with the result of lymphocytes count shown in Fig. 2e. Of note, the positive regulation of apoptotic process signal was activated while the pyroptosis signal was inhibited, indicating that the type of cell death caused by LL and HH irradiation appeared to be apoptosis instead of pyroptosis (Fig. 6a and b). Furthermore, the apoptosis signal was more enriched in the LL group (Fig. 5d), which was consistent with the TUNEL results (Fig. 4f, g).

Furthermore, we focused on the differential expression genes that exhibited changes specifically after LL irradiation or exclusively after HH irradiation. The enrichment results of the genes exclusively upregulated in LL groups showed that the amino acid metabolism and transport, pyrimidine ribonucleoside triphosphate biosynthetic process, positive regulation of protein processing et al. were activated (Fig. 6c). Neutrophil degranulation was also enriched in these genes set, consistent with the results of neutrophils count shown in Fig. 2d. In addition, the genes exclusively downregulated in the LL groups could be enriched in several immune associated signals, including production of molecular mediator of immune response, adaptive immune response, acute inflammatory response to antigenic stimulus, defense response to bacterium, which suggests that the immune signals were inhibited after LL irradiation (Fig. 6d).

Furthermore, the enrichment results of the genes exclusively upregulated in HH groups showed that the signaling pathways of positive regulation of cell migration, vasculature development, extracellular matrix organization, etc. were activated (Fig. 6e). It’s worth noting that immune associated responses, such as positive regulation of cytokine production, immune effector process, and chemotaxis regulation were exclusively activated after HH irradiation instead of LL irradiation, which indicated that HH could promote immune response and potentially serve as an adjunct to cancer immunotherapy. The HH exclusive negative genes were enriched in the signals of the electron transport chain, amino acid metabolic process, mitochondrion organization et al. (Fig. 6f). Notably, the signals of amino acid metabolism and biosynthesis were inhibited upon HH irradiation but activated after LL irradiation. Besides, the mitochondrial organization, biogenesis and metabolism, and biological oxidation were inhibited upon HH irradiation. Mitochondrial activity is associated with ROS production and energy metabolism (Limoli and Vozenin 2023). The inhibition of mitochondrial activity upon HH irradiation may reduce the superoxide production and decrease the biological oxidation, which prevents damage to biomacromolecules. Besides, the genes of eicosanoid metabolism via lipoxygenases LOX signaling pathway were exclusively downregulated upon HH irradiation (Fig. 6f), suggesting reduced lipid oxidation in this condition.

### HH irradiation increased macrophage infiltration in the intestine

**Fig. 7:**
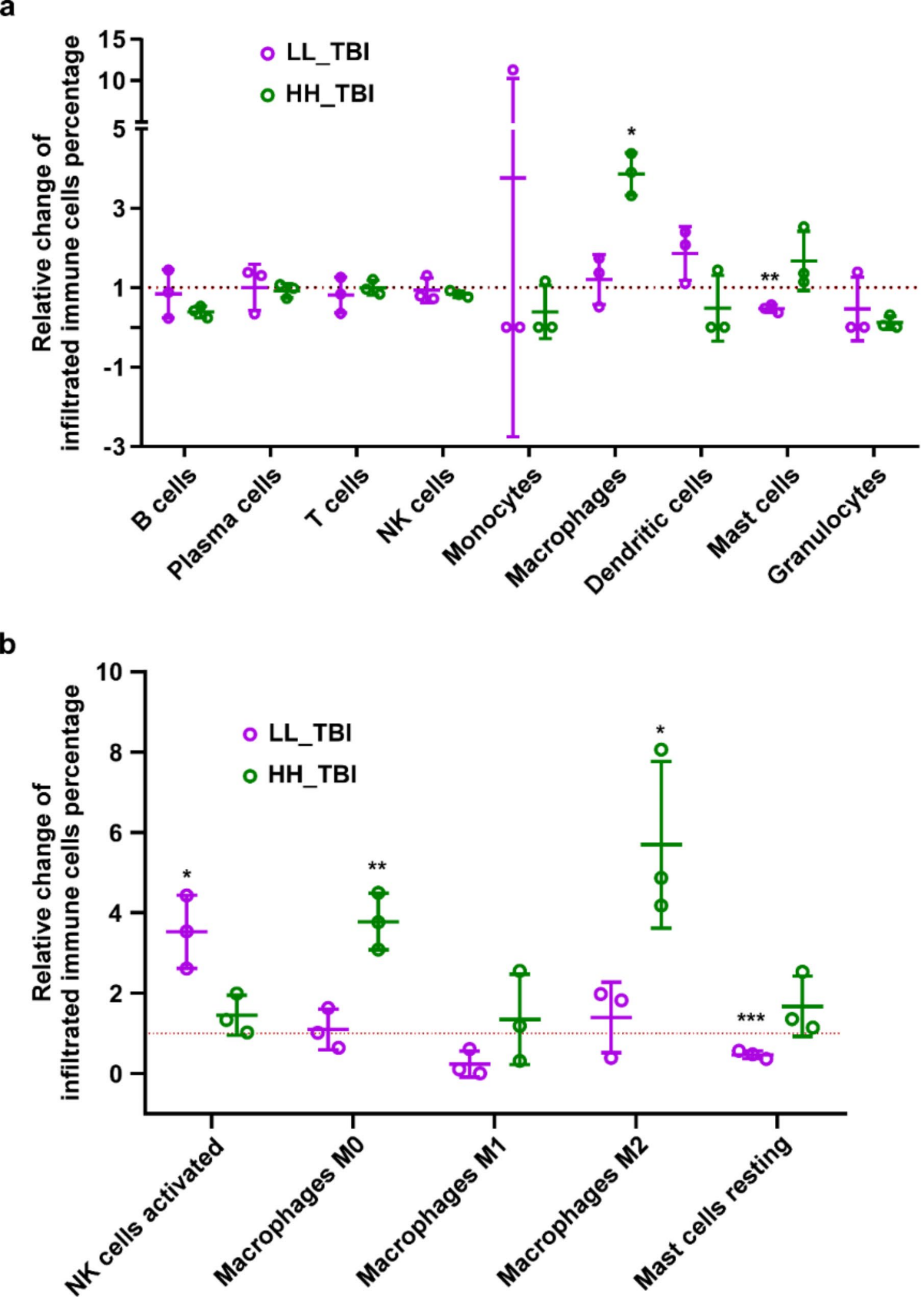
Analysis of immune cells infiltration by CIBERSORTx. (**a**) Relative change of infiltrated immune cells percentage in the intestinal tissue after LL and HH TBI, utilized transcriptome sequencing for CIBERSORTx analysis. (**b**) Relative change of significantly altered infiltrated immune cell percentages, including NK cells activated, Macrophages M0/M1/M2, and Mast cells resting. Each group consist of 3 samples, * *p* < 0.05, ** *p* < 0.01 vs. control

To investigate the activated immune response upon HH irradiation instead of LL irradiation, we utilized the RNA-seq result analyzed with the CIBERSORTx tool to assess the infiltration of immune cells in the intestine after LL or HH irradiation (Fig. 7). The mean percentages of immune cells infiltrated in three samples of each group were shown with Fig. 7. The results indicate that the relative percentage of activated natural killer (NK) cells increased, while resting mast cells decreased after LL irradiation. These results suggest that LL irradiation enhanced immune activation. Moreover, infiltrations of macrophages M0 and M2 were increased upon HH irradiation (Fig. 7b). M2 macrophages can promote cell proliferation and tissue repair, as well as secrete anti-inflammatory cytokines, which provide a potential explanation for the sparing effect of HH irradiation. In conclusion, LL irradiation triggers a shift toward immune activation (NK cells), while HH irradiation appears to promote tissue repair and anti-inflammatory responses (M2 macrophages).

## Discussion

The unique biological response of UHDR radiation in sparing normal tissue has emerged with great research interest. However, the optimal parameter combination for UHDR radiation remains unclear. Moreover, due to the challenges in producing large field high dose rate beams, studies on the ultra-high dose rate TBI are very limited (Hornsey et al. 1971, Smyth et al. 2018. In this study, different dose rate combinations were adopted to comprehensively explore the biological outcomes following TBI. HH and LH mode irradiations were performed using a compact linear accelerator, and the instantaneous dose rate reached over 10^5^ Gy/s for the HH and LH mode irradiation, while the mean dose rates were 468 Gy/s and 0.4 Gy/s for HH and LH mode irradiation, respectively. Conventional LL mode irradiations were performed using a clinical LINAC. We investigated mortality, acute hematologic toxicity, inflammatory cytokine response, and intestinal damage after 10 Gy TBI. Our results indicate that instantaneous dose rate has a pivotal role in UHDR radiation, and when the instantaneous dose rate is sufficiently high (> 10^5^ Gy/s), both ultra-high or low mean dose rate irradiation (HH and LH mode) can reduce the mortality after 10 Gy TBI (Fig. 2). Generally, a mean dose rate of 40 Gy/s is considered as the threshold to trigger FLASH sparing effect. (Zhang et al. 2023) and Bell et al. (Bell et al. 2024) used proton beams over 100 Gy/s and observed no sparing effects, highlighting that mean dose rate alone may not fully predict FLASH outcomes. This underscores the importance of other beam parameters, e.g., instantaneous dose rate, which our study further supports. Besides, a recent study by Dai et al. demonstrated that a single 20 Gy UHDR pulse (200 Gy/s, 0.1s delivery time) and 10 × 2 Gy UHDR pulses (200 Gy/s, 10 min total delivery time) both reduce lung tissue damage (Dai et al. 2023). These findings suggest that the FLASH sparing effect may not be constrained by a strict 1-second delivery time as proposed by (Montay-Gruel et al. 2021). Further research is needed to better define the UHDR parameter thresholds necessary for optimizing radiotherapy outcomes.

Our results reveal a substantial increase in neutrophil count at 3 h and 48 h post irradiation, accompanied by a significant decrease in white blood cell and lymphocyte counts 48 h post irradiation (Fig. 2). Neutrophils are essential components of the innate immune response and a major contributor to inflammation (Christopher and Link 2007). The early increase in neutrophil count may be attributed to rapid recruitment of these cells from the bone marrow, leading to an inflammatory cytokine response with increased IL-6 and TNF-α levels in the serum. At the acute stage of exposing to radiation, inflammation plays a crucial role in the repair of tissue damage. However, the persistence of inflammation return to be detrimental in the late stage post irradiation (Bell et al. 2019). Our results indicate that more intense pro-inflammatory and anti-inflammatory responses were observed at 3 h after HH and LH mode irradiation (Fig. 3). The pro-inflammatory levels were downregulated at 48 h after HH and LH mode irradiation, consistent with our previous results demonstrating that the inflammatory cytokines response is strong immediately after UHDR radiation but it can readily return to the unirradiated control level (Zhu et al. 2022). In contrast, the inflammatory response remained elevated at 48 h (Fig. 3a and b) post conventional LL irradiation, and persisted at high levels until 6 weeks post irradiation (Zhu et al. 2022).

Bell B I et al. reported that a single fraction of 18 Gy focal irradiation targeted at intestine induces significant infiltration of innate and adaptive immune cells localized to the irradiated area, and leads to significantly increased serum IL-6 level, IL-6 signaling blockade exacerbates acute and late intestinal injury (Bell et al. 2019). Previous results from Grivennikov S et al. suggest that IL-6 produced by lamina propria myeloid cells protects normal intestinal epithelial cells from apoptosis (Grivennikov et al. 2009). Our TUNEL assay results indicated that intestinal apoptosis was significantly reduced in the HH and LH group (Fig. 4d, f), this could result from the protective effect of high-level IL-6 secretion since 3 h post irradiation. In contrast, the inflammatory response was elevated from 3 h to 48 h post LL irradiation, and could perpetuate for even 6 weeks, as indicated by our previous results (Zhu et al. 2022), this could lead to late intestinal tissue damage.

To investigate the mechanisms of FLASH sparing effect in the intestine, our study utilized transcriptome sequencing for analyzing the differentially expressed genes between LL and HH modes of irradiation. Both modes of irradiation can promote cell migration, wound healing, degradation of the extracellular matrix, cell adhesion, inflammatory response et al. Lymphocyte activation was inhibited at both LL and HH modes, which represented the myelosuppression after irradiation. The signals of amino acid metabolism and protein processing were activated at the LL mode, while inhibited at the HH mode. In addition, the neutrophil degranulation was exclusively activated upon LL irradiation, which is consistent with the result of blood counts (Fig. 2d). The immune associated signals are activated after HH irradiation, while inhibited upon LL irradiation. The protection of immune cells could potentially enhance the efficacy of tumor immunotherapy, our study suggested the potential benefit for the combination of immunotherapy and UHDR radiotherapy. We also utilized CIBERSORTx to analyze the infiltration of immune cells in the intestinal tissues, and found that M2 macrophage infiltration upon HH irradiation may help explain its protective or sparing effect on tissues, as these macrophages are associated with healing and anti-inflammatory cytokine secretion. However, the presence of M2 macrophages in a tumor microenvironment raises concerns: M2 macrophages can facilitate tumor progression by promoting cell proliferation and tissue remodeling. This dual role emphasizes the need for caution when interpreting their increased presence, as it could also pose a risk for tumor growth in specific contexts. The reduction in resting mast cells and the activation of NK cells after LL irradiation highlight an immune-activating effect, potentially beneficial for targeting abnormal cells but possibly leading to more inflammatory damage compared to HH irradiation. This highlights the complexity of irradiation’s impact on the immune microenvironment and the need for careful consideration of irradiation levels based on therapeutic goals, especially in oncological settings.

Vascular injury induced by radiotherapy is an important part of radiation injury. The current evidence only supports that UHDR radiotherapy results in less vascular damage than CONV-RT, but the impact of UHDR radiotherapy on the upstream gene regulatory pathway is not clear (Lin et al. 2022). We found that the vasculature development was activated upon HH irradiation, indicating that the vasculature repair occurred more promptly following UHDR-induced vascular injury (Fig. 6e). We also found that the phosphorylated H2AX after HH irradiation was lower compared to LL irradiation (Fig. 4b, c). In addition to initialization of DNA repair, the cellular radiation response involves slowing and arrest of the cell cycle to prevent cells with DNA damage lesions from replicating (Friedl et al. 2022). The signal of cell cycle checkpoints was enriched by exclusively downregulated genes in the HH group (Fig. 6e), which aligns with reduced DNA damage repair and continued cell cycle progression after HH irradiation. Mitochondria serve as the compartment for the oxidative respiratory chain, and the structural and functional integrity of mitochondria is closely linked to the production of ROS (Limoli and Vozenin 2023; Lin et al. 2022). The downregulation of mitochondrial biogenesis and metabolism is associated with the reducing of biological oxidations and DNA damages, contributing to normal tissue resistant to UHDR radiation. Taken together, our finding summarized the differential genes expression between LL and HH irradiation modes, and provided comprehensive explanations for the mechanism underlying the ultrahigh dose rate irradiation sparing effect in normal tissue.

Our results indicated that the ultra-high instantaneous dose rate (HH and LH modes) irradiation TBI reduced mortality and healthy tissue toxicities compared to conventional LL irradiation, this suggests the instantaneous dose rate is a crucial parameter for triggering the FLASH sparing effect. Ideally, the high mean dose rate and low instantaneous dose rate (HL) irradiation mode should be investigated. However, achieving high mean dose rate irradiation requires the accelerator to operate at a high duty cycle, resulting in extreme thermomechanical stress that can overwhelm a compact linear accelerator. The use of a superconducting accelerator could be an option to reduce thermomechanical stress and enable more flexible dose rate combinations, and this awaits further achievements in the UHDR accelerator development.

## Conclusions

Our study emphasizes the crucial role of instantaneous dose rate in triggering the UHDR TBI sparing effects. We investigated the mortality, hematologic toxicity, inflammatory cytokine response, intestinal damage, and differential gene expression after 10 Gy TBI with different dose rate combinations. We found that when the instantaneous dose rate is sufficiently high (> 10^5^ Gy/s), both ultra-high and low mean dose rate irradiation (HH and LH mode) reduced mice mortality, myelosuppression, DNA damage, and cell apoptosis. The results of transcriptome sequencing provide comprehensive explanations for the mechanism of HH sparing effect in normal tissue. The activation of immune associated responses, after HH irradiation may provide further potential anticancer benefits, while suppression of mitochondrial oxidation-related processes and increased infiltration of M2 macrophages might explain the normal tissue-sparing effect of UHDR irradiation.

## Electronic supplementary material

Below is the link to the electronic supplementary material.

Supplementary Material 1

Supplementary Material 2

Supplementary Material 3

Supplementary Material 4

## Data Availability

Research data are stored in an institutional repository (https://www.researchdata.org.cn/, RDDB2024627309) and will be shared upon request to the corresponding author.
