# Supplementary material for: Instantaneous dose rate as a crucial factor in reducing mortality and normal tissue toxicities in murine total-body irradiation: a comparative study of dose rate combinations
Source: Mol Med. 2025 Feb 26;31:79. doi: 10.1186/s10020-025-01135-3 (PMC11866584; doi:10.1186/s10020-025-01135-3)
Supplement: Supplementary file 1 — Supplementary Material 1 [file 10020_2025_1135_MOESM1_ESM.docx]

**Supplementary**


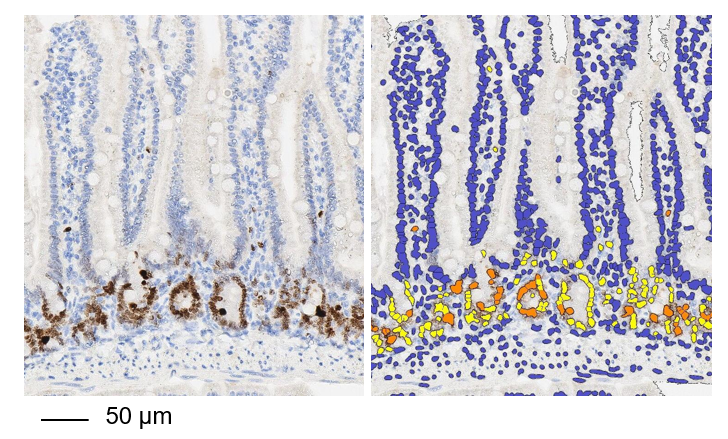


**Figure S1. Illustration of image analysis using HALO platform.** Left. Original scanned ki67 stained section. Right. marked Ki67 negative (blue marks), moderate positive (yellow marks), and strong positive (orange marks) cells.
